# COVID-19 and Acute Coronary Syndromes: From Pathophysiology to Clinical Perspectives

**DOI:** 10.1155/2021/4936571

**Published:** 2021-08-30

**Authors:** Luca Esposito, Francesco Paolo Cancro, Angelo Silverio, Marco Di Maio, Patrizia Iannece, Antonio Damato, Carmine Alfano, Giuseppe De Luca, Carmine Vecchione, Gennaro Galasso

**Affiliations:** ^1^Department of Medicine, Surgery and Dentistry, University of Salerno, Baronissi, Salerno, Italy; ^2^Department of Chemistry and Biology, University of Salerno, Fisciano, Italy; ^3^Vascular Pathophysiology Unit, IRCCS Neuromed, Pozzilli, Isernia, Italy; ^4^Division of Cardiology, Azienda Ospedaliera-Universitaria “Maggiore della Carità, ” Eastern Piedmont University, Novara, Italy

## Abstract

Acute coronary syndromes (ACS) are frequently reported in patients with coronavirus disease 2019 (COVID-19) and may impact patient clinical course and mortality. Although the underlying pathogenesis remains unclear, several potential mechanisms have been hypothesized, including oxygen supply/demand imbalance, direct viral cellular damage, systemic inflammatory response with cytokine-mediated injury, microvascular thrombosis, and endothelial dysfunction. The severe hypoxic state, combined with other conditions frequently reported in COVID-19, namely sepsis, tachyarrhythmias, anemia, hypotension, and shock, can induce a myocardial damage due to the mismatch between oxygen supply and demand and results in type 2 myocardial infarction (MI). In addition, COVID-19 promotes atherosclerotic plaque instability and thrombus formation and may precipitate type 1 MI. Patients with severe disease often show decrease in platelets count, higher levels of d-dimer, ultralarge von Willebrand factor multimers, tissue factor, and prolongation of prothrombin time, which reflects a prothrombotic state. An endothelial dysfunction has been described as a consequence of the direct viral effects and of the hyperinflammatory environment. The expression of tissue factor, von Willebrand factor, thromboxane, and plasminogen activator inhibitor-1 promotes the prothrombotic status. In addition, endothelial cells generate superoxide anions, with enhanced local oxidative stress, and endothelin-1, which affects the vasodilator/vasoconstrictor balance and platelet aggregation. The optimal management of COVID-19 patients is a challenge both for logistic and clinical reasons. A deeper understanding of ACS pathophysiology may yield novel research insights and therapeutic perspectives in higher cardiovascular risk subjects with COVID-19.

## 1. Introduction

After the identifications of the first cases in Wuhan, China, coronavirus disease 2019 (COVID-19) rapidly spread worldwide and took on pandemic proportions [1, 2].

Although primarily affecting the respiratory tract, the clinical course of COVID-19 may be complicated by several systemic and potentially life-threatening conditions, with a reported inhospital mortality rate ranging from 9% to 15% [3].

Cardiovascular (CV) involvement is frequently reported in COVID-19 and may impact on patient clinical outcome and mortality risk [4–7].

Acute coronary syndrome (ACS) has been reported in a substantial proportion of patients with COVID-19 [8, 9]. Although the underlying pathogenesis remains unclear, several potential mechanisms have been hypothesized, including direct viral cellular damage, systemic inflammatory response with cytokine-mediated injury, microvascular thrombosis, endothelial dysfunction, and oxygen supply/demand imbalance due to the severe hypoxic state [10–14]. Moreover, as described in other infective diseases, COVID-19 may promote atherosclerotic plaque instability and thrombus formation and precipitate type 1 myocardial infarction (MI) [15, 16].

In this review, we aimed at describing the pathophysiological mechanisms of ACS in patients with COVID-19, with a focus on the translational perspectives and potential clinical applications.

## 2. Pathogenesis and Transmission of COVID-19

COVID-19 is caused by severe acute respiratory syndrome coronavirus-2 (SARS-CoV-2), a novel *β*-coronavirus infecting human cells of the respiratory tract, vascular endothelium, heart, gut, and immune system [17]. The virus binds the angiotensin-converting enzyme 2 (ACE2) receptor, highly expressed on the target host cells, through a spike (S) protein that enables the fusion of membranes and viral internalization [18, 19]. In particular, endothelial cells and cardiac pericytes express abundant ACE2, making them highly susceptible to SARS-CoV-2 interaction and internalization.

The interferon-mediated upregulation of ACE2 may facilitate the involvement of adjacent pneumocytes and the development of an uncontrolled inflammatory reaction, microvascular thrombosis, interstitial and alveolar edema, and eventual progression toward acute respiratory distress syndrome (ARDS) [20], Moreover, it has been hypothesized that SARS-CoV-2, by interacting with ACE2 for the cell entry, causes a downregulation of the bound ACE2 and increases the circulating level of soluble ACE2. This deregulation affects the activity of bound ACE2, which is associated with several beneficial effects by regulating the inflammatory response, reducing oxidative stress, and promoting vessel relaxation via the production of angiotensin1-7 (Ang1-7) [17, 21]. It seems reasonable to hypothesize that, similarly to SARS-CoV-1, SARS-CoV-2 promotes the cleavage of ACE2 receptors leading to lower Ang1-7 serum levels [22]. However, only one in vitro study has showed that SARS-CoV-2 downregulates the ACE2 expression, and further studies are needed to confirm this pathophysiological pathway [23]. SARS-CoV-2 is transmitted from person to person via close contact through respiratory droplets and viral particles inhalation, with a mean incubation of about five days [24]. Viral load detected in the asymptomatic and symptomatic subjects appears to be similar, suggesting that asymptomatic subjects can transmit the virus as well as the symptomatic ones [25].

The initial symptoms are very similar to other viral respiratory syndromes and include fever, cough, shortness of breath, fatigue, myalgias, headache and gastrointestinal involvement. [1] The clinical spectrum of COVID-19 manifestations is particularly wide, ranging from asymptomatic or minimally symptomatic to life-threatening or fatal forms, characterized by systemic inflammatory response syndrome, ARDS, multiple organ failures, and death [26]. CV involvement has been frequently reported in hospitalized patients with COVID-19 and may impacts the length of hospitalization, clinical severity, rate of admission in intensive care unit, and probability of survival [4, 9].

## 3. The Epidemiology Paradox of ACS in COVID-19

Although COVID-19 may be complicated by coronary plaque instability and myocardial oxygen supply/demand imbalance, multiple investigators worldwide have reported a marked reduction in the rate of hospitalization for ACS during the peak of pandemic. Data from the ISACS-STEMI COVID-19 registry showed a significant drop in the number of ST-elevation MI (STEMI) patients invasively treated from 2019 to 2020, with a 18.9% reduction of admissions for STEMI in the last year. Patients treated in 2020 also had longer ischemia and door-to-balloon time [27]. Another study conducted in China showed that the number of primary percutaneous coronary intervention (PCI) dropped by more than a half in the first three months of 2020 compared to 2018 and 2019, while the number of patients treated with fibrinolysis increased by 2 to 3 times. Also in this study, a longer time to reperfusion was reported among patients treated in 2020 [28].

The significant reduction of hospitalization for ACS registered all over the world has been associated with a substantial reduction in the total number of urgent and emergent coronary angiography performed [29–33]. Despite the delay in the reperfusion time, most of STEMI patients without COVID-19 underwent emergent coronary angiography and primary PCI as per standard of care [34]. Conversely, COVID-19 patients with STEMI frequently did not receive guideline-recommended treatments, and the use of fibrinolysis over PCI has been reported in a high number of cases [27, 28, 35, 36]. In a cohort of 78 COVID-19 patients with STEMI, noninvasive treatment with fibrinolysis, instead of primary PCI, was reported as the most performed strategy in 3 out of 4 cases [27].

Changes in the epidemiology of STEMI may have several potential interpretations. First, social distancing, the fear of contagion, and the prominent media attention on the uncontrolled spread of the disease might have reduced the awareness of the population towards other life-threatening conditions, such as ACS. Second, the redistribution of healthcare resources in the struggle against the overwhelming pandemic could have weaken the local emergency networks, as highlighted by the reported system delays. Additionally, although seemingly contradictory given the risk of COVID-19-related thrombotic complications, the contribution of an actual reduction of ACS due to less emotional and physical triggering cannot be excluded, a plausible phenomenon after the prompt adoption of national lockdowns during the first period of the pandemic [37].

The lower rate of admission for STEMI has been associated with the increased incidence of out-of-hospital cardiac arrest and mechanical complications reported in this period [38–40]. This has raised the attention of health organizations worldwide and calls for caution and further investigations.

## 4. Mechanisms of ACS in COVID-19

**Figure 1:**
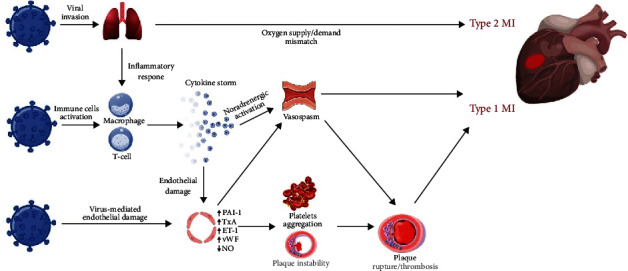
Pathophysiology of ACS in COVID-19. Mechanisms involved in the pathophysiology of ACS in patients with COVID-19. SARS-CoV-2, by binding the ACE2 receptors expressed on the surface of the host cell, may infect pneumocytes, macrophages, and endothelial cells. The respiratory impairment related to the pulmonary involvement, ranging from pneumonia to ARDS in severe forms, causes hypoxia and type 2 MI due to the oxygen supply/demand mismatch. Also, the infection promotes an aberrant inflammatory response resulting in the release of cytokines and proinflammatory molecules such as IL-1, IL-6, IL-7, TNF*α*, and IFN*γ*. Cytokines have the potential to damage the endothelial function with increased production of oxidative stress agents and prothrombotic factors. SARS-CoV-2 may also exert a direct cellular effect by interacting with molecules expressed on the surface of the endothelial cells. In turn, the inflammatory environment enhances the instability of preexisting atheromatous plaques, promotes platelets activation and aggregation, and upregulates the sympathetic nervous system resulting in increased vasomotility and coronary spasm. The interplay of all these mechanisms may favor plaque rupture and thrombosis leading to type 1 MI. ACS: acute coronary syndrome; ACE2: angiotensin-converting enzyme 2; COVID-19: coronavirus disease 2019; IFN*γ*: interferon *γ*; IL-1: interleukin 1; IL-6: interleukin 6; IL-7: interleukin 7; MI: myocardial infarction; SARS-CoV-2: severe acute respiratory syndrome coronavirus 2; TNF*α*: tumor necrosis factor *α*.

The potential underlying mechanisms of ACS in COVID-19 may be multiple and to date are not fully understood. The spectrum of pathophysiological mechanisms reflects the distinctive clinical features of patients with confirmed MI diagnosis, such as the angiographic evidence of non-obstructed coronary arteries, stent thrombosis, multiple thrombotic culprit lesions, and high thrombus burden [27, 41, 42]. Sometimes, MI has been the first manifestation of the disease, suggesting that ACS should be considered as a specific thrombotic complication of SARS-CoV-2 infection [41, 43]. The most recognized mechanisms include cytokine-mediated systemic inflammatory response, prothrombotic activation of the coagulation cascade, endothelial dysfunction, and hypoxic injury due to oxygen supply/demand imbalance (Figure 1).

### 4.1. Hemostatic Abnormalities

Several hemostatic abnormalities have been reported in COVID-19. Patients with severe disease often show decrease in platelets count, higher levels of d-dimer, and prolongation of prothrombin time [44, 45]; these abnormalities have showed negative prognostic impact in cohorts of hospitalized patients with COVID-19. In an observational study conducted in Wuhan, China, patients admitted to the intensive care unit (ICU) had significant higher plasma levels of d-dimer than patients who did not need ICU care [46]. Similarly, Tang et al. reported increased levels of d-dimer and fibrinogen degradation products and a mild prolongation of prothrombin time in fatal cases, compared with patients who survived [47]. The reason for this prothrombotic state is not completely understood; moreover, it is unclear whether these abnormalities are imputable to direct viral effects on the coagulation cascade or to the cytokine-mediated inflammatory response [48]. A recent prospective study comparing laboratory findings in cases with COVID-19-related ARDS with a historic cohort of patient with nonCOVID-19-related ARDS showed a significant increase in procoagulant factors in patients with SARS-CoV-2 infection, correlated with the elevation of the acute phase reactants. These findings suggest a major role of the cytokine storm (CS) in COVID-19-related coagulopathy [49]. Cytokines produced during the systemic inflammatory response induce the overexpression of ultralarge von Willebrand factor multimers (ULVWF) and tissue factor (TF), which are involved in the primary and secondary hemostatic mechanisms, respectively [47, 50, 51]. These factors may act as major triggers in the activation of the coagulation cascade, resulting in a hypercoagulability status characterized by increased production of thrombin [52]. In addition, the presence of a positive lupus anticoagulant (LA) might further contribute to SARS-CoV-2- related coagulopathy [50]. LA antibodies are produced in clinical circumstances characterized by high cellular lysis, such as infectious, inflammatory, and immune diseases; in such cases, the occurrence of cellular damage, caused by the oxidative stress on the endothelium, exposes phospholipids usually not accessible to the immune system, with consequent induction of thrombus formation [53].

This multifactorial coagulopathy justifies the common incidence of life-threatening thrombotic complications, such as venous thromboembolism (VTE), pulmonary embolism (PE), and ACS [7, 10, 14, 41]. More specifically, patients with ACS and concurrent COVID-19 represent a distinctive clinical setting characterized by hallmarks of heightened thrombogenicity. Choudry et al. compared COVID-19 patients with STEMI with a control group of SARS-CoV-2-negative STEMI and reported a higher incidence of multiple thrombotic culprit lesions, higher thrombus grade, and lower rate of procedural success of primary PCI procedures as assessed by myocardial blush grade (a marker of myocardial perfusion) [42, 54]. Notably, high coronary thrombus burden and low myocardial blush grade were associated with higher d-dimer plasma levels. Eventually, in a population of 91 COVID-19 patients with STEMI, Rodriguez-Leor et al. reported a high rate of stent thromboses (4.1%) [30], a potentially catastrophic event with lower than 1% incidence at one year in contemporary STEMI cohorts [55, 56].

### 4.2. Endothelial Dysfunction

Vascular endothelium is a central interface between circulatory apparatus and tissues and plays a key role in vascular homeostasis. A functional endothelium possesses several valuable properties for regulating vasomotion, inflammation, platelet reactivity, coagulation, vascular permeability, and host defense. Traditional CV risk factors such as diabetes, hypertension, older age, and smoking may damage the endothelium through several mechanisms, including oxidative stress related to the increased intracellular levels of superoxide anions. All these mechanisms shift towards a vasoconstrictive and procoagulant status typical of the dysfunctional endothelium, which is a distinctive feature of patients with coronary artery disease (CAD) [57, 58].

Recent findings indicate that endothelial dysfunction represents one of the most detrimental mechanisms of COVID-19 pathophysiology [59, 60]. The endothelial injury may be induced by both direct viral effects, as demonstrated by the presence of viral elements within the endothelium and inflammatory cell accumulation, resulting in venous, arterial, and microvascular thrombosis [13, 61]. Several pathways seem to be involved in the development of endothelial-mediated complications of COVID-19. While in physiological conditions, the endothelium maintains anticoagulant, antithrombotic, and profibrinolytic characteristics, when stimulated by inflammatory and infectious triggers, it can shift toward an opposite array of functions through the expression of tissue factor, the release of von Willebrand factor (vWf), and the production of thromboxane and plasminogen activator inhibitor-1 (PAI-1) [62–64]. Under normal conditions, a functional endothelium is able to limit oxidative stress, a recognized contributor to the progression of atherosclerosis, through the expression of superoxide dismutase and glutathione [65, 66]. In contrast, when activated by inflammatory cytokines, endothelial cells generate superoxide anions with consequent enhancement of local oxidative stress, which has been associated with a higher risk of MI and other CV consequences [67–69]. The enhanced production of endothelin-1, a potent vasoconstrictor and prothrombotic agent, may also favor the vasodilator/vasoconstrictor imbalance, platelet aggregation and, finally, myocardial ischemia [70]. Recent findings from a single-centre study also reported higher levels of thrombomodulin, a specific marker of endothelial injury, which resulted associated with poor inhospital outcome [60].

Another pathway of endothelial dysfunction may be vascular endothelial glycocalyx (VEGLX) SARS-CoV-2-mediated damage. VEGLX is composed by glycosylated lipid-protein mixtures that covers vascular endothelium and plays an important role in maintaining vascular homeostasis [71]. A variety of conditions, including inflammatory response, hypoxia, hyperglycemia, and ischemia/reperfusion injury, are known to be associated with VEGLX damage through several degradation pathways [72–74]. Moreover, high circulating levels of VEGLX components are associated with poor outcomes in critically hill patients [75]. Severe COVID-19 forms represent the typical scenario in which glycocalyx damage might occur. Several reports observed high concentrations of VEGLX injury biomarkers in patients hospitalized for COVID-19, including syndecan-1, hyaluronic acid, and sTie-2 [75–78]. Moreover, Stahl et al. found a severe depletion of heparanase-2, an enzyme with protective effects on VEGLX structure and functions [76].

Despite the paucity of data, it is possible to hypothesize that VEGLX damage might contribute to the progression of endothelial dysfunction in severe COVID-19, with anticipated consequences on the development of thrombotic and vascular complications.

All these mechanisms might be amplified in case of preexisting endothelial dysfunction, such as in patients with CV risk factors and CAD, leading to a heightened risk of ACS and other thrombotic complications. Advances in the understanding of SARS-CoV-2-related endothelial dysfunction, beyond the pathophysiological insights, would encourages the assessment of the utility of pharmacological therapies targeting the endothelium, such as ACE-inhibitors (ACEi) and statins, in large prospective randomized studies [79–82].

### 4.3. Inflammatory Response and Cytokine Storm

Immune and inflammatory response is chronically involved in the progression of atherosclerotic disease. [83] In the context of viral infection, the inflammation may spoil the regular homeostasis and trigger a prothrombotic state by activating platelets and promoting endothelial dysfunction [15]. Moreover, infections can increase the sympathetic activity with consequent vasoconstriction in the coronary tree [84]. The interplay between all these biological and mechanical conditions can induce atheromatous plaque erosion or rupture, resulting in coronary thrombosis and ACS [85, 86].

The clinical course of severe forms of COVID-19 is characterized by an aberrant inflammatory response and CS [46, 48]. CS is a process primed by the primordial inflammatory cytokine interleukin-1 (IL-1), which has the ability to induce its own gene expression and to endorse a self-powered inflammatory response [87]. IL-1 stimulates the production of other proinflammatory mediators such as tumor necrosis factor (TNF*α*), interleukin-6 (IL-6), and chemoattractant molecules, involved in tissue penetration of inflammatory cells [88–90]. In addition to the local effects, IL-6 induces the synthesis of acute phase reactants, such as fibrinogen, plasminogen activator inhibitor-1 (PAI-1), and favor a prothrombotic and antifibrinolytic imbalance. SARS-CoV-2-related CS is confirmed by several studies showing increased level of proinflammatory factors, such as IL-1, IL-6, IL-10, IFN*γ*, granulocyte colony stimulating factor (G-CSF), monocyte chemoattractant protein (MCP1), macrophage inflammatory protein 1 alpha (MIP1A), platelet-derived growth factor (PDGF), TNF*α*, and vascular endothelial growth factor (VEGF) [46, 91]. The extensive production of proinflammatory cytokines disrupts the physiological homeostasis guaranteed by the functional endothelium, thus contributing to thrombosis and local tissue injury [47, 50, 92]. The pathophysiological relationship between COVID-related CS and thrombotic events suggests the implementation of targeted therapy for prevention and treatment of ACS in this setting [93].

### 4.4. Oxygen Supply/Demand Imbalance

Hypoxemic respiratory failure is the leading cause of death in COVID-19, accounting for nearly 60% of cases with fatal outcome [94]. The severe hypoxic state, combined with other mechanisms observed in COVID-19, such as sepsis, tachyarrhythmias, anemia, hypotension, and shock, can induce a myocardial damage due to the mismatch between oxygen supply and demand in absence of atherothrombotic lesions, findings consistent with the diagnosis of type 2 MI [95]. Compared with type 1 MI, patients with type 2 MI show distinct clinical features and poorer prognosis, largely related to the higher prevalence of coexisting systemic diseases [96]. Given their high complexity and vulnerability, critically ill patients with COVID-19 are particularly prone to the occurrence of type 2 MI, which strongly contributes to the reported high rate of inhospital mortality [4].

## 5. Myocardial Infarction with Nonobstructive Coronary Arteries

Myocardial infarction with nonobstructive coronary arteries (MINOCA) has been frequently reported in patients with COVID-19 [30, 97]. In an Italian series of 28 STEMI patients with COVID-19, 11 patients (39.3%) did not have obstructive CAD [43]. In another series of 18 COVID-19 patients with STEMI from New York, coronary angiography did not detect obstructive CAD in 33% of cases [41]. Several mechanisms have been proposed for these cases, including plaque erosion, microthrombi, or coronary vasospasm [41, 98, 99]. The pathophysiology seems to be multifactorial and encompasses inflammatory activation, oxidative stress, and endothelial dysfunction in the context of COVID-19-related CS [100, 101]. The underlying mechanisms of MINOCA, albeit theoretical, are largely underinvestigated due to difficulties in performing invasive and noninvasive diagnostic work-up including intravascular imaging, pharmacological provocative test, and cardiac magnetic resonance [102–104].

Takotsubo syndrome (TTS), a condition that simulates an ACS at presentation, has been frequently reported during COVID-19 pandemic [105]. Observational studies on hospitalized patients with COVID-19 and laboratory evidence of myocardial injury have estimated an incidence of TTS ranging from 2% to 4% of cases [9, 106, 107]. Although TTS has been proposed as a direct manifestation of COVID-19, it may be also the consequence of the physical and emotional stress related to the SARS-CoV-2 infection leading to sympathetic overdrive [108]. A higher risk of TTS has also been described in the context of pandemic, irrespective of SARS-CoV-2 infection. In a cohort study of consecutive patients admitted for suspected ACS at Cleveland Clinic, TTS diagnosis was reported in 7.8% of patients during the first wave of the pandemic, being significantly higher than in prepandemic timelines [109]. These findings have been considered the consequence of growing psychological stress related to the pandemic context (e.g., fear of contagion, social distancing, and isolation), but need confirmation in larger prospective studies [110].

## 6. Therapeutic Perspectives

The optimal management of ACS in COVID-19 patients is a demanding challenge, both for logistic and clinical reasons. During the first wave of the pandemic, in order to reduce healthcare workers exposure and the risk of contagion, several scientific societies suggested the use of fibrinolysis as first-line therapy in STEMI patients with COVID [111–113]. However, the delay in reperfusion time, the increased risk of mortality and left ventricular dysfunction, and frequent absence of a coronary culprit lesion do not seem to support such an approach as an alternative to the guideline-recommended treatment with primary PCI and optimal antithrombotic therapy, currently considered as the standard of care [34, 42, 114].

Antithrombotic therapy in COVID-19 is an active area of investigation, with multiple ongoing randomized clinical trials evaluating a variety of regimens with antiplatelet, anticoagulant, or their combinations (Table 1). Antiplatelet agents, besides being the cornerstone of pharmacological treatment of ACS, may deserve a remarkable role in the setting of COVID-related endothelial injury and thromboinflammation. Their anti-inflammatory and antithrombotic effects, indeed, provide the pathophysiological rationale for a systematic use in this clinical setting. Activated platelets release several inflammatory mediators, such as cytokines, chemokines, and metalloproteinases, further contributing to the sustainment of a systemic inflammatory response [115]. Moreover, the interaction between activated platelets and neutrophils induces neutrophil activations, extracellular matrix protein degradation, and prothrombotic endothelium activation [116, 117].

Several antiplatelet agents, such as glycoprotein IIb/IIIa antagonists and P2Y12 inhibitors, also showed protective effects on lung injury in patients with viral respiratory infections, due to the limitation of neutrophil recruitment [118]. Although all P2Y12 inhibitors have the potential to reduce platelet–leukocyte aggregates and platelet-derived proinflammatory cytokines, ticagrelor is unique in the inhibition of ENT1 (equilibrative nucleoside transporter 1), resulting in higher antiplatelet, but also antibacterial activity [119]. Previous data from non-COVID-19 cohorts showed the reduction of lung injury and the prevention of septic complications in patients with pneumonia treated with ticagrelor, resulting in a significant survival benefit [120, 121]. However, to counterbalance the well-documented risk of thrombotic complications, diffuse alveolar hemorrhage was reported as a possible autoptic finding in COVID-19 and severe acute respiratory syndrome [122, 123]. The extensive thrombosis of pulmonary capillary bed, if left uncontrolled, leads to secondary fibrinolysis, consumption coagulopathy, disseminated intravascular coagulation, and diffuse alveolar hemorrhage. Also, considering the high bleeding risk profile of frail patients hospitalized for severe COVID-19, some authors hypothesized the possibility of a shorter duration dual antiplatelet therapy after an ACS [124, 125].

Thus, the selection of the proper antiplatelet regimen in COVID-19 patients with ACS remains an open clinical issue; it should be based on the careful assessment of ischemic and bleeding risk and tailored to the individual patient.

Anticoagulant agents might yield clinical benefits in COVID-19, not only for their antithrombotic action but also for additional anti-inflammatory effects. Unfractionated heparin (UFH) and low-molecular-weight heparin (LMWH) have pleiotropic anti-inflammatory effects, including the inhibition of the interaction between platelets and neutrophils and the reduction of the release of inflammatory mediators like IL-1*β*, IL-6, E-selectin, and ICAM-1 [126–128]. Heparins have also direct antiviral effects mediated by their interaction with heparan sulfate, a component of cell surface identified as the initial contact point between human cells and several viruses, including SARS-CoV-2 [129–131]. Heparins compete with heparan sulfate and hamper the interaction between virus and target cells.

Additional antiviral effects of heparin may be related to the interaction with SARS-CoV-2S proteins. Each S protein is characterized by two subunits, S1 and S2; S1 presents the binding domain which interacts with ACE2 receptor on host cells. Heparin has been demonstrated to bind the S1 subunit, causing a structural change that jeopardizes the viral entry mechanism into the target cells. [129] After the interaction with ACE2, the cleavage of S1-S2 subunits is needed to expose S2 for adhesion of cell membrane and to finalize the virus entrance into the target cell. This step is permitted by several proteases, including factor Xa, cathepsin, thrombin, and furin. The anticoagulant agents that inhibit these proteases, including heparins and oral anticoagulants, might exert antiviral effects due to the interference with SARS-CoV-2 in host cell infection.

Although the pathophysiology of COVID-19 seems to provide a rationale for the use of anticoagulant drugs in routine clinical practice, also in patients without clinically evident thrombotic complications, there are still controversial data on what is the best anticoagulant agent and on the optimal dosing [132, 133]. An observational study on 4,389 patients showed a lower rate of mortality in patients treated with anticoagulants; there was no incremental benefit of therapeutic over prophylactic regimens, but an increased risk of bleeding in patients treated with therapeutic doses [134]. Results from randomized clinical trials, currently ongoing, are urgently needed to clarify these controversies. In the meantime, the choice of the optimal anticoagulation strategy should be individually considered, particularly in patients with COVID-19 and life-threatening thrombotic complications, like ACS [135, 136]. The optimal antithrombotic therapy in COVID-19 patients with ACS and indication for chronic oral anticoagulation therapy (e.g., atrial fibrillation and mechanical valvular prosthesis) is a poorly explored clinical scenario. The choice of type and duration of antithrombotic therapy should be tailored on patients' characteristics taking into account the trade-off between ischemic and bleeding risks, which varies on an individual basis. Due to the higher risk of thrombotic events, some authors have suggested a more aggressive treatment in COVID-19 patients at higher risk of thromboembolic events, such as those with an indication for combined antiplatelet and anticoagulation therapy. However, there are no evidence supporting such an approach and to date seems reasonable to follow the general recommendations proposed for patients with ACS and indication for concomitant oral anticoagulation.

The progressive understanding of COVID-19 pathophysiology provides us a conceptual framework for treatment of endothelial dysfunction. ACEi and statins, two widely used drugs in CV diseases, have robust evidence on their ability to improve endothelial function [80, 82].

The renin-angiotensin-aldosterone system, a key regulator of vascular homeostasis, effectively participates in COVID-19 CV manifestations. Given the central role of ACE2 in COVID-19 pathophysiology, its interaction with SARS-CoV2 might represent a potential therapeutic target [17, 19]. ACE2, unlike ACE, is not antagonized by ACEi, and its expression and activity provide beneficial effects through different pathways [137]. Most of these properties are related to the production of angiotensin1-7, a molecule with multiple anti-inflammatory, antioxidant, vasodilatory, and natriuretic effects, exerted through the G-protein coupled receptor Mas [138]. Ang1-7 production can be obtained by several mechanisms: ACE2 can convert angiotensin II (Ang II) directly into Ang1-7 via C-terminus cleavage, representing the most efficient way of production of Ang1-7. Ang1-7 can also be produced from the Ang I via an alternative pathway mediated by the metallopeptidase neprilysin. In addition, ACE2 can convert Ang I into the Angiotensin1-9 (Ang1-9) intermediate, which itself is converted into Ang1-7 by ACE; however, the latter two pathways are less efficient [137]. Conversely, SARS-CoV-2, similarly to other coronaviruses, limits ACE2 expression by promoting its cleavage by the specialized proteinase A disintegrin and metalloproteinase 17 (ADAM17), leading to a reduction in ACE2's protective effects [22]. Previous studies showed that ACEi increases the transcription of ACE2 mRNA and plasma levels of Ang1-7, therefore suggesting an ACEi-mediated upregulation of the ACE2/Ang1-7/Mas pathway [139]. In light of this evidence, the use of ACEi might lead to anti-inflammatory, antifibrotic, and antithrombotic effects, providing a valuable protection against ACS in patients with COVID-19.

Statins, beyond their effect on circulating lipids, exert pleiotropic effects on the immune response by modulating immune cell adhesion and migration, antigen presentation, and cytokine production. These effects are mediated by their capability of inhibiting the production of small GTPases (Ras, Rho, Rac) and modulate the plasma level of the myeloid differentiation primary response 88 (MYD88), leading to the downregulation of inflammatory transcriptional factors, like NF-*κΒ* [140, 141]. Statins also reduce reactive oxygen species and increase the production of antioxidants, restoring the normal endothelial function and integrity by upregulating eNOS and consequently increasing the production of NO by the endothelium [142]. Statin-mediated NO production has been associated with a reduction of platelets reactivity; [143] additional antithrombotic properties of statins are related to the inhibition of thromboxane A2 and isoprostane formation, the downregulation of TF production, and the increase of thrombomodulin levels [144]. Like ACEi, statins can upregulate the expression of ACE2 via epigenetic histone modification, favoring the beneficial effects of the upregulation of the ACE2/Ang1-7/Mas axis [145]. This wide range of pharmacodynamic properties would support the use of statins in COVID-19, aiming to antagonize the proinflammatory and prothrombotic endothelial modifications of the disease [146]. A recent propensity-matched observational study on 13,981 patients has showed a reduction in all-cause mortality in patients hospitalized for COVID-19 and treated with statins [147]. Albeit, these findings from general COVID-19 population need confirmation by large randomized clinical trials, and the rationale for the use of statins would be stronger in patients with multiple risk factors for ACS.

Beta-blockers (*β*-blockers) are widely used in different cardiovascular diseases, including ACS. They have been proposed in patients with COVID-19 to antagonize the disease-related hyperinflammatory response [148, 149]. In fact, beta2-adrenergic receptors are widely expressed on immune cells such as macrophages, dendritic cells, and T and B lymphocytes and seem to play a relevant role in promoting macrophage activation and proinflammatory cytokine production (IL-6, TNF-*α*, and NF*κΒ*) [150–153].

Since CS is involved in the pathogenesis of vascular complications in COVID-19, the reduction of circulating cytokines driven by *β*-blockers could mitigate their systemic detrimental effects. The rationale for the use of *β*-blockers is reinforced in COVID-19 patients who develop left ventricular systolic dysfunction and heart failure (HF) after STEMI. Rodriguez-Leor et al. reported a higher percentage of HF on admission in STEMI patients with vs. those without COVID-19 (31.9% vs. 18.4%) [30]. Consistently, Choudry et al. showed that left ventricular ejection fraction after PCI was lower in STEMI patients with COVID-19 than in those without (42.5% vs. 45%) [42]. The potentially beneficial anti-inflammatory and cardiac effects of *β*-blockers need to be confirmed by large multicenter studies.

## 7. Conclusions

Despite the overall reduction in cases admitted at the emergency departments during the early phase of the pandemic, ACS is a potential life-threatening complication of COVID-19. The pathophysiological mechanisms are multiple and include atherosclerotic plaque rupture, overactivation of the coagulation system, platelet hyperreactivity, abnormal systemic inflammatory response, and oxygen supply/demand imbalance. When compared to non-COVID-19 cases, patients with ACS and SARS-CoV-2 infection present distinctive clinical and anatomical features including the absence of obstructive CAD, the higher burden of thrombus, and the angiographic evidence of multiple thrombotic lesions. Deeper understanding of the ACS pathophysiology in COVID-19 may allow the application of translational notions in daily clinical practice. The use of pharmacological agents, namely, antiplatelets, anticoagulants, ACEi, *β*-blockers, and statins, seems a valuable strategy not only in the treatment of ACS but also as a preventive strategy in higher CV risk subjects with COVID-19.

## Figures and Tables

**Table 1:** Ongoing randomized clinical trials investigating antithrombotic regimens in patients with COVID-19.

Study name	Registration number	Population	Treatments	Design	Estimated enrollement (*n*)	Primary endopoint	Time of FU (days)
PARTISAN	NCT04445623	Non-ICU patients	Prasugrel 10 mg	Randomized, double blind	128	P/F ratio	7
PEAC	NCT04365309	Non-ICU patients	Aspirin 100 mg	Randomized, open label	128	Clinical recovery time The time of SARS-CoV2 overcasting	14 37
ACT-COVID19	NCT04324463	Non-ICU patients	Aspirin Rivaroxaban Colchicine	Randomized, open label, factorial	4000	Colchicine vs. control; aspirin and rivaroxaban vs. control: (i) Composite of invasive mechanical ventilation or death (ii) Disease progression of 2 points on a 7-point scale Aspirin and rivaroxaban vs. control: (i) Composite of MACE (MI, stroke, acute limb ischemia, VTE, death)	45
C-19-ACS	NCT04333407	Non-ICU patients	Aspirin 75 mg Clopidogrel 75 mg Rivaroxaban 2.5 mg Atorvastatin 40 mg Omeprazole 20 mg	Randomized, open label	3170	All-cause mortality	30
RESIST	CTRI/2020/07/026791	Non-ICU patients	Aspirin 75 mg Atorvastatin 40 mg	Randomized, open label	800	Clinical deterioration expressed as progression of WHO clinical improvement ordinal score ≥ 6	10
COVID-PACT	NCT04409834	ICU patients	UFH iv enoxaparin 1 mg/kg Clopidogrel 75 mg UFH sc Enoxaparin 40 mg/0.4 mL	Randomized, open label, factorial	750	Hierarchical composite: death due to venous or arterial thrombosis, pulmonary embolism, clinically evident DVT, type 1 MI, ischemic stroke, systemic embolism, or acute limb ischemia or clinically silent DVT	28

COVID-19: coronavirus disease 2019; DVT: deep vein thrombosis; ECMO: extra corporeal membrane oxygenation; ICU: intensive care unit; MACE: major adverse cardiovascular events; MI: myocardial infarction; P/F: PaO2/FiO2; RRT: renal replacement therapy; SARS-CoV2: severe acute respiratory syndrome coronavirus 2; UFH: unfractionated heparin; VTE: venous thromboembolism.
